# FYCO1 Increase and Effect of Arimoclomol–Treatment in Human *VCP*–Pathology

**DOI:** 10.3390/biomedicines10102443

**Published:** 2022-09-30

**Authors:** Anne-Katrin Guettsches, Nancy Meyer, René P. Zahedi, Teresinha Evangelista, Thomas Muentefering, Tobias Ruck, Emmanuelle Lacene, Christoph Heute, Humberto Gonczarowska-Jorge, Benedikt Schoser, Sabine Krause, Andreas Hentschel, Matthias Vorgerd, Andreas Roos

**Affiliations:** 1 Department of Neurology, Heimer Institute for Muscle Research, University Hospital Bergmannsheil, Ruhr University Bochum, 44789 Bochum, Germany; 2 Department of Neuropediatrics and Neuromuscular Centre for Children and Adolescents, Center for Translational Neuro- and Behavioral Sciences, University Duisburg–Essen, 45147 Essen, Germany; 3 Manitoba Centre for Proteomics and Systems Biology, 715 McDermot Aveue, Winnipeg, MB R3E 3P4, Canada; 4 Department of Internal Medicine, University of Manitoba, 820 Sherbrook Street, Winnipeg, MB R3A 1R9, Canada; 5 Department of Biochemistry and Medical Genetics, University of Manitoba, 745 Bannatyne Avenue, Winnipeg, MB R3E 0J9, Canada; 6 Leibniz–Institut für Analytische Wissenschaften—ISAS—e.V, 44227 Dortmund, Germany; 7 Nord/Est/Ile–de–France Neuromuscular Reference Center, Unité de Morphologie Neuromusculaire, Institute of Myology, Pitié–Salpêtrière Hospital, APHP, Sorbonne University, 75013 Paris, France; 8 Department of Neurology, Medical Faculty, Heinrich–Heine–University Düsseldorf, 40225 Düsseldorf, Germany; 9 Department of Neurology, Friedrich–Baur–Institute, Ludwig–Maximilians–University Munich, Ziemssenstr. 1a, 80336 Munich, Germany; 10 Children’s Hospital of Eastern Ontario (CHEO) Research Institute, Ottawa, ON K1H 5B2, Canada

**Keywords:** valosin–containing protein (VCP), inclusion body myopathy with frontotemporal dementia and Paget’s disease of the bone (IBMPFD), fibroblast proteomics, arimoclomol, autophagy, FYCO1, GBP1, DDX58

## Abstract

Dominant *VCP*–mutations cause a variety of neurological manifestations including inclusion body myopathy with early–onset Paget disease and frontotemporal dementia 1 (IBMPFD). *VCP* encodes a ubiquitously expressed multifunctional protein that is a member of the AAA+ protein family, implicated in multiple cellular functions ranging from organelle biogenesis to ubiquitin–dependent protein degradation. The latter function accords with the presence of protein aggregates in muscle biopsy specimens derived from *VCP*–patients. Studying the proteomic signature of *VCP*–mutant fibroblasts, we identified a (pathophysiological) increase of FYCO1, a protein involved in autophagosome transport. We confirmed this finding applying immunostaining also in muscle biopsies derived from *VCP*–patients. Treatment of fibroblasts with arimoclomol, an orphan drug thought to restore physiologic cellular protein repair pathways, ameliorated cellular cytotoxicity in *VCP*–patient derived cells. This finding was accompanied by increased abundance of proteins involved in immune response with a direct impact on protein clearaqnce as well as by elevation of pro–survival proteins as unravelled by untargeted proteomic profiling. Hence, the combined results of our study reveal a dysregulation of FYCO1 in the context of *VCP*–etiopathology, highlight arimoclomol as a potential drug and introduce proteins targeted by the pre–clinical testing of this drug in fibroblasts.

## 1. Introduction

Valosin–containing–protein (VCP) is ubiquitously expressed in human tissues and contributes to different cellular functions including modulation of autophagy, maturation of autophagosomes, control of the ubiquitin–proteasome system (UPS), and endocytosis [1,2]. In the context of modulation of apoptosis and autophagy, VCP has also been linked to the regulation of cellular survival [2].

Disorders associated with genetic defects of *VCP* are characterized by a considerable phenotypic variability [3,4], which might accord with the functional spectrum of the corresponding protein. Thus, due to its numerous cellular functions, the clinical presentation of pathogenic variants is complex and may involve several tissues such as skeletal muscle, neurons or bones. However, *VCP*–associated disorders are rare, which in combination with the great variety of clinical presentations complicates a correct and rapid diagnosis [3]: The most frequent *VCP*–associated disorder is inclusion body myopathy (IBM) associated with Paget’s disease of the bone (PDB) and frontotemporal dementia (FTD), a symptomatic trio leading to the term IBMPFD [4,5,6,7]. Moreover, other neurological manifestations like amyotrophic lateral sclerosis (ALS) [8], progressive spastic paraplegia [9], distal myopathy [10], facio–scapulo–humeral weakness [11], parkinsonism [6], Charcot–Marie–Tooth disease [12] and Huntington’s disease [13] have been described in association with pathogenic *VCP*–variants. Due to the numerous tissues involved and the diverse clinical presentations associated with *VCP*–related disorders, the term “multisystem proteinopathy (MSP)” has recently emerged to summarize, among others, disorders associated with pathogenic variants affecting the *VCP*–gene [14].

Notably, detailed analysis of skeletal muscle tissue derived from patients with sporadic inclusion body myositis (sIBM) revealed an overlap of pathophysiological processes with *VCP*–associated MSP. Both muscular diseases are characterized by the presence of rimmed vacuoles on the morphological level, which are known to contain proteins playing different roles in autophagy/protein degeneration, such as p62, LC3, several chaperones, TDP–43 and VCP [6,15]. Moreover, FYCO1 (FYVE and coiled–coil domain–containing protein 1), an autophagic adaptor protein and binding partner of p62, has been shown to be increased in rimmed vacuoles of patients with sIBM [15,16]. Further hints toward a common pathophysiology are given by the fact that rare missense variants in *VCP* and *SQSTM1* have been identified in patients with sIBM [4,15,17].

Based on the pathophysiological similarities (especially regarding p62/SQSTM1), protein dysregulations common in sIBM and *VCP*–related myopathy have also been studied by investigating muscle tissue derived from *Vcp*–mutant mice presenting with a vacuolar myopathy [18]. In this context, the effect of arimoclomol, an agent that co–induces the heat–shock response, also has been investigated in *Vcp*–mutant mice, revealing an improvement of grip strength and of the toe spreading reflex after treatment [18]. Based on these promising findings, the effect of arimoclomol has been investigated as a potential medication in a clinical trial in sIBM patients but remained without a clinical benefit [18]. However, the pre–clinical results on the *Vcp*–mutant mice published by Ahmed and co–workers suggest a better benefit for *VCP*–patients.

To further explore pathophysiological processes shared between sIBM and *VCP*–related myopathy, we here investigated the abundance and distribution of FYCO1 in quadriceps biopsies and fibroblasts derived from genetically confirmed *VCP*–patients. Given that cultured skin fibroblasts have been described to serve as a suitable model in the study of the etiopathology of rare neurological diseases [19], we therefore utilized this *VCP in vitro* model to study the effect of arimoclomol application in human–derived material by focussing on the identification of new biochemical targets of this drug.

## 2. Materials and Methods

### 2.1. Muscle MRI on VCP–Patients

Patients were scanned in a 1.5T scanner with a 4–channel phased–array coil (Magnetom Symphony; Siemens, Munich, Germany) using a standardized protocol (T1–weighted spin–echo [repetition time (TR)/echo time (TE) 500/20 ms, slice thickness 10 mm, matrix 512 3 512], 2 T2–weighted short tau inversion recovery [TR/TE 4020/68 ms and 3040/27 ms, respectively, inversion time 150 ms, slice thickness 10 mm, matrix 512 3 512]).

### 2.2. Fibroblasts and Cell Culture

Fibroblasts as a suitable in vitro model to study the molecular etiology of neurological diseases [19] were isolated from fresh donor skin biopsies following standardised EuroBioBankprotocols (www.eurobiobank.org/biobanking–sops (accessed on 1 June 2021)): biopsies were washed with sterile phosphate–buffered saline (PBS) and digested at 37 °C for 15 min with 2.5% trypsin (Thermo Fisher Scientific, Darmstadt, Germany) and a further 90 min with 0.5% collagenase (Type IV, Sigma–Aldrich, Taufkirchen, Deutschland). Fibroblasts were proliferated in Ham’s F–10–Complete Medium (Thermo Fisher Scientific, Darmstadt, Germany) supplemented with 20% foetal bovine serum (FBS, SeraLab—BioIVT, West Sussex, UK), 2% penicillin–streptomycin (Thermo Fisher Scientific, Darmstadt, Germany), 1% GlutaMAX™ (Thermo Fisher Scientific, Darmstadt, Germany) and 1% fungizone (Thermo Fisher Scientific, Darmstadt, Germany). Once fibroblast cells attained sufficient confluency, they were frozen and stored long–term in liquid nitrogen. Fibroblasts derived from *VCP*–patients were obtained from the MRC Centre Neuromuscular Biobank. For further experiments including proteomic profiling, cells were cultured as described above until a confluence of 70%, harvested by scraping from culture flasks and washed twice with ice–cold PBS; cell pellets were then snap–frozen in liquid nitrogen and stored at −80 °C until further processing. A concentration of 10 µM arimoclomol (dissolved in ddH_2_O) was supplemented for 16 h.

### 2.3. Proteomic Profiling on Human Skin Fibroblasts

Human fibroblast cell pellets of three biological replicates from healthy (control) and three biological replicates derived from patients with a recurrent *VCP*–mutation (p.Arg155His) were lysed in 6 M GuHCl, 50 mM ABC, pH 7.8, followed by sonication with an ultrasonic probe (20% duty cycle, 1 × 20 s, 2 s pulse). Cysteines were reduced with 10 mM DTT for 20 min at 56 °C and alkylated with 30 mM IAA for 20 min in the dark, at room temperature. In the meanwhile, a BCA (Thermo Scientific, Darmstadt, Germany) was used to determine protein concentrations. Samples were diluted with 50 mM ABC, pH 7.8, to a final concentration of 0.5 M GuHCl. Next, samples were pre–heated for 5 min at 56 °C, followed by digestion with trypsin (Promega, Walldorf, Germany, sequencing grade) in a ratio of 20:1 (sample:enzyme) overnight at 37 °C in the presence of 2 mM CaCl_2_. Digestion was quenched by acidification with TFA to a final concentration of 1%. Before LC–MS measurement, samples were spun down for 1 min at 18,000× *g* and an aliquot corresponding to 1 µg of peptide was diluted to 15 µL with 0.1% TFA and immediately analyzed by nano–LC–MS/MS on an Orbitrap Fusion Lumos online coupled to an Ultimate 3000 nano RSCL equipped with a 100 μm × 2 cm C18 Acclaim Pepmap trapping column and a 75 μm × 50 cm C18 Acclaim Pepmap main column, both maintained at 60 °C. Samples were pre–concentrated on the trap column in 0.1% TFA for 5 min at a flow rate of 20 µL/min followed by separation on the main column at a constant flow rate of 250 nL/min using a binary buffer (A: 0.1% formic acid; B: 84% acetonitrile, 0.1% formic acid) ranging from 7–42% B in 52 min. MS scans were acquired from *m*/*z* 300–1500 at a resolution of 120,000 with an automatic gain control (AGC) of 2 × 10^5^ and a maximum injection time of 50 ms. The 15 most intense ions were selected for data dependent acquisition (HCD) with an isolation window of m/z 0.8 and an AGC of 5 × 10^4^ and fragmented with a normalized higher energy collisional dissociation (HCD) energy of 34; MS/MS scans were acquired in the orbitrap with a resolution of 15,000. The dynamic exclusion was set to 30 s. 

Label free quantification was performed with the Progenesis QI for proteomics software version 3.0.6039.34628 from Nonlinear Dynamics (Newcastle upon Tyne, UK). MS raw files were automatically aligned, followed by peak picking. Only MS/MS spectra with ranks 1–10 were exported as peak lists with a limited fragment ion count of 100 and using deisotoping and charge deconvolution. Using searchGUI 3.59 [20], spectra were searched against a target/decoy version of the human Uniprot database (downloaded September 2014, containing 20,194 target sequences) using X!Tandem Vengeance (2015.12.15.2) [21]. Additionally, spectra were searched using Mascot 2.4.1 (Matrix Science). Search parameters were: “trypsin” as enzyme with a maximum of 2 missed cleavage, carbamidomethylation of Cys set as fixed modification, oxidation of Met set as variable modification, MS and MS/MS tolerances of 10 ppm and 0.02 Da, respectively. PeptideShaker 1.4.010 was used to combine all search result files and filter data at an FDR of 1% on the protein and peptide level. Obtained identifications were exported using the advanced PeptideShaker features that allow direct re–import of the quality–controlled data into Progenesis.

Only proteins quantified with at least two unique peptides were exported from Progenesis. Then, for each protein, the average of the normalized abundances (obtained from Progenesis) from the biological replicate analyses was used to calculate log2 ratios and fold–changes using Perseus. Only proteins with an adjusted *p*–value < 0.05 were considered as significantly regulated.

The same approach was applied to identify protein targeted by arimoclomol by investigating the above–mentioned patient–derived fibroblasts treated (10 µM) and non–treated with arimoclomol.

### 2.4. Immunostaining on Quadriceps Muscle Biopsies

Immunostaining on quadriceps muscle biopsies derived from *VCP*–patients and controls were carried out as described previously [15]. For that purpose, the following antibodies were used: anti–FYCO1 (Abcam, Cambridge, UK; ab224152; 1:100) and anti–p62 (Abcam, Cambridge, UK; ab109012; 1:100).

### 2.5. Immunostaining on Fibroblasts

Immunostaining on cultured skin fibroblasts derived from *VCP*–patients and controls were carried out as described previously [19]. For that purpose, the following antibodies were used: anti–FYCO1 (Abcam, Cambridge, UK; ab224152; 1:100), anti–p62 (Abcam, Cambridge, UK; ab109012; 1:100), anti–VCP (Genetex, Eching, Germany; GTX113030; 1:100), anti CD63 (Abcam, Cambridge, UK; ab8219; 1:100) and anti–tenascin (Thermo Fisher Scientific, Darmstadt, Germany; MA5–16086; 1:100).

### 2.6. Analyses of Cellular Fitness on Arimoclomol Treated Fibroblasts

To address the effect of arimoclomol on the fitness of *VCP*–mutant fibroblasts, an MTT–assay was carried out as described previously [22]. For that purpose, fibroblasts from two controls and three *VCP*–patients were analysed (eight replicates per sample) with and without supplementation of 10 µM arimoclomol (see above).

## 3. Results

### 3.1. Clinical Findings

Skeletal muscle MRI of the calves of two representative *VCP*–patients (patient 1 and patient 2 described in Table 1) showed slight edema and fatty degeneration of the right anterior muscles, the left medial gastrocnemius and the soleus muscles in patient 1 (Supplementary Figure S1). Patient 2 showed more severe changes on muscle MRI, with muscle edema pronounced of the right calf muscles and moderate to severe fatty degeneration of the gastrocnemius and soleus muscles, more pronounced of the gastrocnemius medialis muscles (Supplementary Figure S1). Neurological symptoms at age of biopsy, age of onset, and muscle biopsy findings are listed in Table 1 along with CK values and molecular genetic information.

### 3.2. Proteomic Signature of Human VCP–Mutant Fibroblasts Revealed Increase of FYCO1

Based on our previous studies, the expression of VCP in cultured human fibroblasts was already confirmed [19]. Proteomic analysis on whole protein extracts of fibroblasts derived from controls and patients with pathogenic *VCP*–variants allowed the quantification of 2219 proteins with at least 2 unique peptides, 358 of which were significantly dysregulated including FYCO1 (1.38–fold increase; log2 ratio) and tenascin/TENA (–1.58–fold increase; log2 ratio) (Supplementary Figure S2).

### 3.3. FYCO1 Is Increased in VCP–Patient Derived Muscle Biopsy Specimen

**Figure 1:**
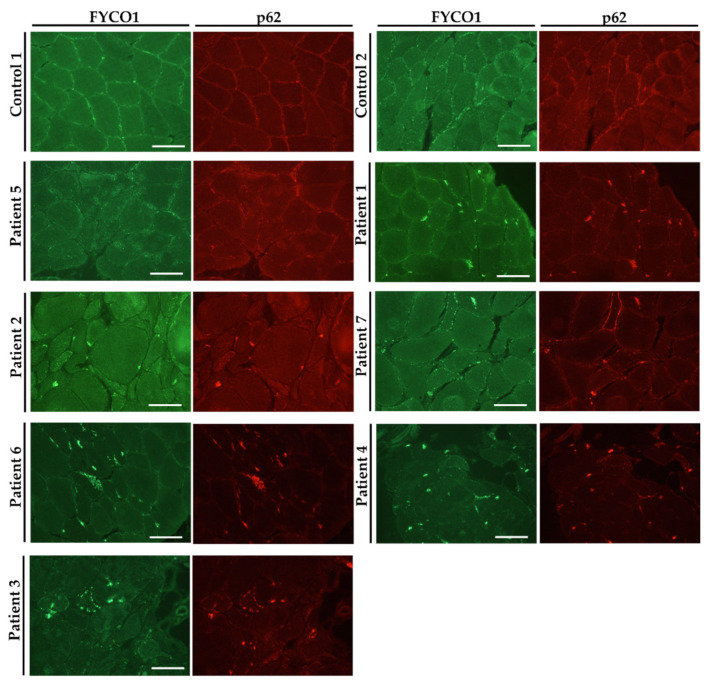
**Immunofluorescence studies on skeletal muscle biopsies derived from *VCP*–patients and controls.** Increase of FYCO1–immunoreactivity (green) within the sarcoplasm and at the subsarcolemmal region which partially overlaps with p62 (red). Images are serialized in succession according to the presence of FYCO1–p62 immunoreactivity. Two representative controls are shown. Scale bars: 100 µm.

To verify our proteomic findings and to study *VCP*–pathology in vitro, immunostaining of FYCO1 along with p62 was carried out on muscle biopsy specimen derived from a total of seven genetically confirmed *VCP*–patients (see Table 1) as well as on four control biopsies. Results of the studies revealed an increased immunoreactivity presenting as sarcoplasmic dots often enriched at sub–sarcolemmal regions in muscle cells derived from *VCP*–patients compared to age–matched controls (two representative controls are shown; Figure 1). These sarcoplasmic dots frequently show a co–immunoreactivity with p62, a known protein aggregation marker and show a pathological appearance ranging from minor (patient 5) to prominent (patient 3) presence (Figure 1). However, in control muscle biopsies, FYCO1–immunoreactivity presented as small dots distributed throughout the sarcoplasm (Figure 1).

### 3.4. Increase of FYCO1 Concomitant with Protein Aggregate Markers and Decrease of Tenascin in VCP–Mutant Fibroblasts

**Figure 2:**
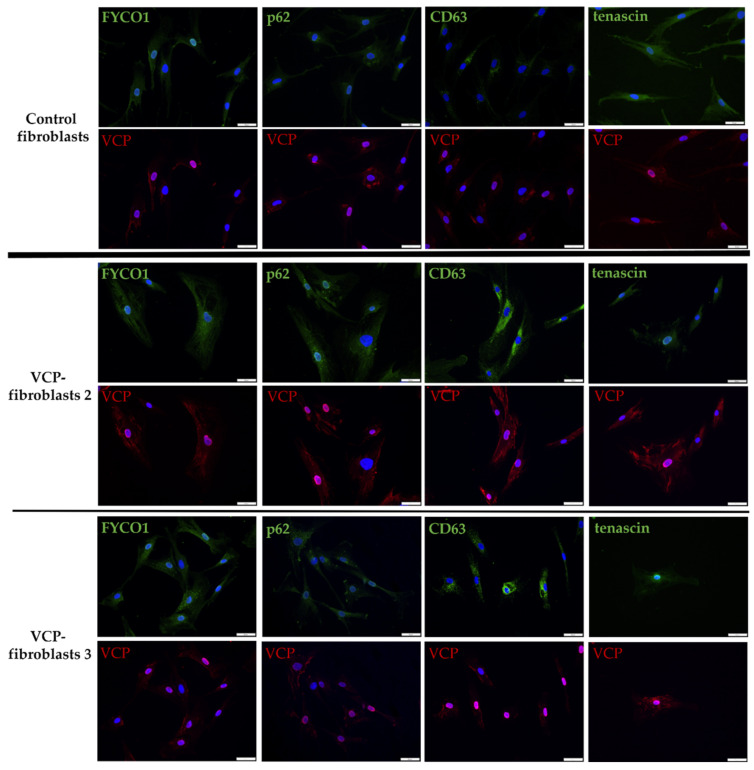
**Immunofluorescence studies on *VCP*–mutant and control fibroblasts with co–staining of VCP (red) and FYCO1, p62, CD63 and tenascin (green).** VCP is increased in the cytoplasm and nuclei of patient derived fibroblasts. CD63 showed a strong cytoplasmic increase in patient fibroblasts compared to controls. FYCO1 and p62 were also more pronounced in patient fibroblasts. Tenascin is reduced in patient–derived fibroblasts. Scale bar = 50 µm. Two representative patients are shown: VCP fibroblasts 2 = patient 2 and VCP fibroblasts 3 = patient 3; patient numbering is consistent with patient numbering in Figure 1.

To confirm the proteomic finding of FYCO1 increase in *VCP*–patient derived fibroblasts and to study *VCP*–pathology in the context of a potential concomitant increase of protein aggregation markers such as FYCO1 and p62 as well as CD63 (a marker of intracellular vesicle transport/phagocytosis) and tenascin (an extracellular matrix protein involved in neuronal migration and regeneration), immunofluorescence studies were carried out. For that purpose, the same fibroblasts as used for the proteomic profiling approach were investigated. Results are displayed in Figure 2 and show a strongly increased amount of CD63–positive perinuclear and cytoplasmic aggregates in patient derived fibroblasts compared to control fibroblasts. FYCO1 and p62 immunoreactivities were also more pronounced in patient fibroblasts. VCP immunoreactivity is more pronounced especially in the nucleus of patient derived fibroblasts (Figure 2). Tenascin presented with reduced immunorectivity in *VCP*–patient derived fibroblasts confirming the proteomic data (Figure 2).

### 3.5. Effect of Arimoclomol–Treatment on the Fitness of VCP–Patient Derived Fibroblasts

**Figure 3:**
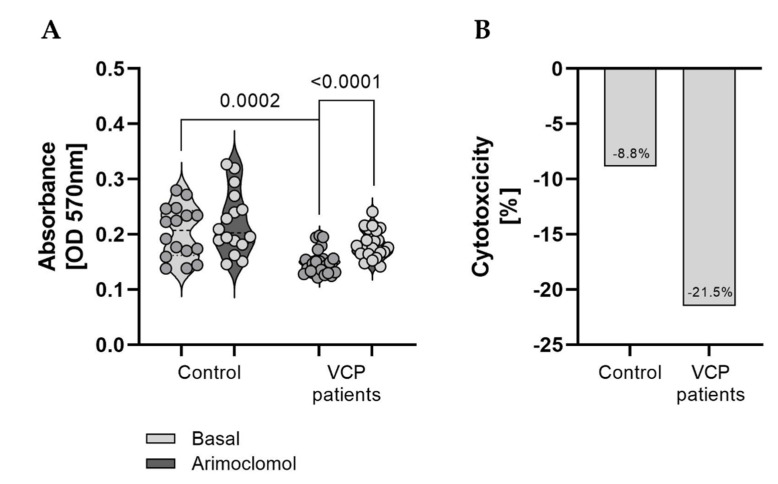
**Studies of cellular fitness on *VCP*–mutant and control fibroblasts.** (**A**) Decrease of proliferation in patient–derived fibroblasts compared to controls under basal conditions. A significant effect of arimoclomol treatment on proliferation of patient–derived cells was observed. Basal fitness levels in control and *VCP*–patient fibroblasts are displayed on the left and fitness levels after treatment with arimoclomol are displayed on the right for each group, respectively. (**B**) Arimoclomol treatment reduced cytotoxicity in patient and control fibroblasts with a higher extend in patient derived cells.

Given that arimoclomol has been shown to be a promising “drug” in a mouse model of *VCP*–pathology [20], we investigated the effect of arimoclomol on the fitness of the fibroblasts derived from *VCP*–patients compared to control cells. For that purpose, an MTT–assay was carried out, which is used to measure cellular metabolic activity as an indicator of cell viability, proliferation and cytotoxicity. This colorimetric assay is based on the reduction of a yellow tetrazolium salt (3–(4,5–dimethylthiazol–2–yl)–2,5–diphenyltetrazolium bromide or MTT) to purple formazan crystals by metabolically active cells. Results of our studies showed a significantly decreased proliferation mirroring a reduced viability in *VCP*–mutant fibroblasts compared to the two grouped controls and a statistically significant increase of proliferation after arimoclomol–treatment in patient–derived fibroblasts whereas the increase after treatment in control cells was not statistically significant (Figure 3A). In addition, results of the MTT–assay displayed a more pronounced increase of viability (mirrored by reduction of cytotoxicity) in *VCP*–mutant fibroblasts treated with arimoclomol compared to treated controls cells (−8.8% versus −21.5%) (Figure 3B).

### 3.6. Effect of Arimoclomol on the Proteomic Signature of VCP–Mutant Fibroblasts

**Figure 4:**
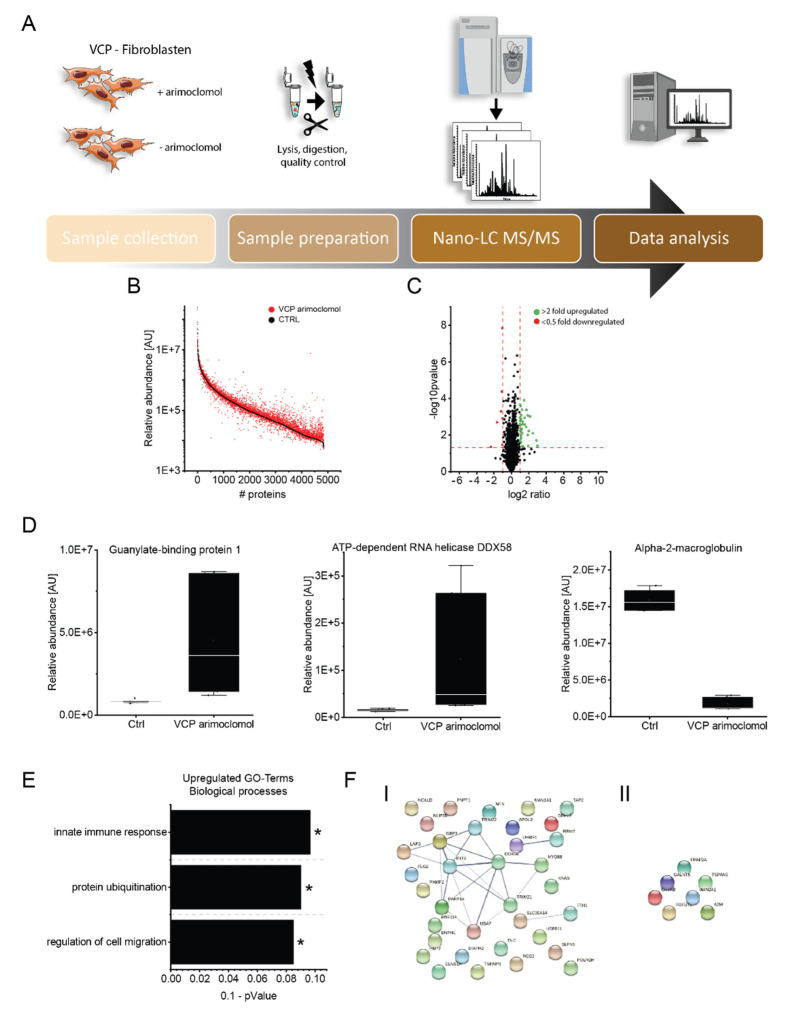
**Effect of arimoclomol treatment on proteostasis of *VCP*–mutant fibroblasts analyzed by proteomic profiling.** (**A**) Workflow applied to biochemically address the effect of arimoclomol on proteostasis of *VCP*–patient derived fibroblasts. (**B**) Abundance plot showing the dynamic range of all identified proteins based on their relative quantification using always the 3 highest abundant peptides for each protein, allowing protein comparison within an experiment. All identified proteins of the control (black) are sorted with decreasing abundance while the patient (red) was plotted in the same order to directly compare the different abundances. All identified proteins cover a dynamic range of eight orders of magnitude. (**C**) Volcano plot highlighting statistically significant increased proteins (green dots) as well as decreased proteins (red dots). (**D**) Box plots of selected dysregulated proteins showing the increase of GBP1, DDX58 as well as decrease of A2MG upon arimoclomol exposure in *VCP*–mutant fibroblasts. (**E**) Results of the GO–term based analysis of affected biological processes based on proteins identified with increased abundance upon arimoclomol treatment of *VCP*–mutant fibroblasts. * indicates a *p*-value ≤ 0.05. (**F**) STRING network analysis of dysregulated proteins separated according to increased (I) and decreased (II).

Given that pathogenic *VCP*–variants lead to characteristic muscle pathology, and a beneficial effect of arimoclomol on muscle pathology was already demonstrated in *Vcp*–mutant mice [18], we aimed to decipher the effect of arimoclomol on proteostasis in fibroblasts derived from *VCP*–patients. By comparing the proteomic signature of cells treated and non–treated with 10 µM arimoclomol for 16 h, we identified a statistically significant increase of 35 and a decrease of 7 proteins (all quantified on the basis of at least two unique peptides). The dysregulated proteins are listed in Table 2 and Figure 4.

Biological processes modulated by the increased proteins include innate immune response, protein–ubiquitination and regulation of cell migration (Figure 4). Notably, among the increased proteins were those with known roles in cellular survival, such as IFIT3 (7.40–fold increased), TFIP8 (3.23–fold increased), tenascin (2.85–fold increased), nestin (2.56–fold increased), RASK (2.25–fold increased) and IFT25 (2.18–fold increased) (Table 2, Figure 4). Arimoclomol treatment also resulted in an increase of proteins involved in immune response such as interferons or cytokines with immune–modulating effects. Notably, some of these immune response proteins are known to have a direct impact on protein clearance by interaction with the ubiquitin–binding protein SQSTM1/p62, which delivers monoubiquitylated proteins to autolysosomes (GBP1: 5.55–fold increased) or on the activation of E3–ubiquitin–protein ligases (DDX58: 8.10–fold increased) (Table 2, Figure 4). Along this line, a subset of E3–ubiquitin–protein ligases is increased in arimoclomol treated *VCP*–mutant fibroblasts even including one for which activity has already been shown to be modulated by interferons (TRI22: 2.97–fold increased) (Table 2, Figure 4). The set of decreased proteins comprises alpha2–macroglobulin (A2MG) (decrease to 0.19–fold abundance) which is able to inhibit all four classes of proteinases by a unique ‘trapping’ mechanism along with proteins involved in glycosylation such as polypeptide N–acetylgalactosaminyltransferase 5 (GALT5) (decrease to 0.50–fold abundance), alpha–mannosidase (MA2A1) (decrease to 0.49–fold abundance) and GDP–fucose protein O–fucosyltransferase 2 (OFUT2) (decrease to 0.47–fold abundance) (Table 2, Figure 4).

### 3.7. Validation of the Effect of Arimoclomol–Treatment on Protein Composition of Fibroblasts Derived from VCP–Patients

**Figure 5:**
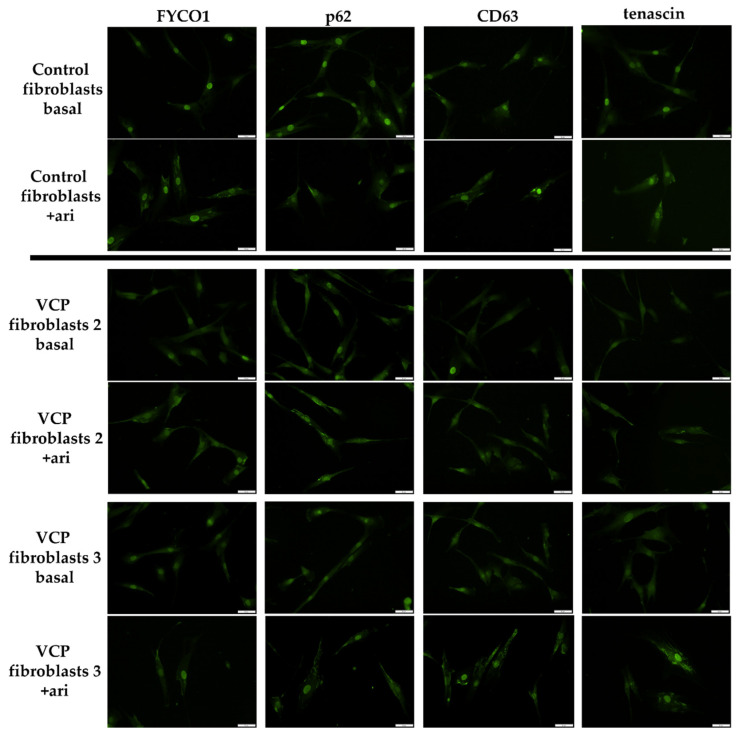
**Immunofluorescence studies on *VCP*–mutant and control fibroblasts at basal conditions and after arimoclomol (ari) treatment.** Arimoclomol treatment resulted in cytoplasmic increase of FYCO1, which is more pronounced in controls (control 1 is shown as one representative control). Decrease of p62–immunoreactivity within the nuclei of control fibroblasts treated with arimoclomol and p62 enrichment within the cytoplasm of *VCP*–mutant fibroblasts. CD63 showed an increased cytoplasmic immunoreactivity in both control and patient derived fibroblasts after arimoclomol treatment. A similar finding was obtained for tenascin. Scale bars = 50 µm. Two representative patients are shown: VCP fibroblasts 2 = patient 2 and VCP fibroblasts 3 = patient 3; patient numbering is consistent with patient numbering in Figure 1.

To verify proteomic data obtained on arimoclomol treated fibroblasts derived from *VCP*–patients, we next performed immunofluorescence studies on arimoclomol exposed cells. Here, we focussed on FYCO1, p62 and CD63 as proteins involved in autophagy–related protein clearance (GBP1 as a p62–binding partner delivering monoubiquitylated proteins to autolysosomes was identified to be increased in treated cells) and tenascin as a pro–survival protein playing crucial roles in synaptic plasticity as well as neuronal regeneration. Our immunofluorescence studies revealed enrichment of FYCO1 in the cytoplasm after arimoclomol treatment. This effect is more pronounced in control than in patient derived fibroblasts (Figure 5). In control fibroblasts treated with arimoclomol, a decrease of p62–immunoreactivity within the nuclei was observed. However, in patient derived fibroblasts, arimoclomol treatment resulted in p62 enrichment within the cytoplasm (Figure 5). After arimoclomol treatment, CD63 showed an increased cytoplasmic immunoreactivity in both control and patient derived fibroblasts (Figure 5). A similar finding was obtained for tenascin (Figure 5).

## 4. Discussion

In the context of our previous study, we demonstrated that fibroblasts serve as a suitable in vitro model to investigate pathophysiological processes related to the manifestation of (rare) genetically caused neurological disorders [19]. Here, we used cultured fibroblasts derived from three *VCP*–patients (all carrying the most recurrent pathogenic variant p.R155H) and applied unbiased proteomic profiling to unravel protein dysregulation providing new insights into the pathophysiology of *VCP*–associated proteinopathy. This approach identified FYCO1, a 1478 amino acid–protein that binds to LC3 and other vesicular cargo and facilitates autophagic degradation as being significantly increased in *VCP*–mutant fibroblasts. Immunofluorescence studies to further characterize this increase of FYCO1 in vitro showed an accompanying increase of VCP, p62 and CD63, which are known modulators of autophagy in p.R155H–*VCP* mutant fibroblasts. This microscopic finding suggests a cellular attempt to increase autophagic flux. However, given that expression of p.R155H–mutant *VCP* has been shown to cause impairment of ubiquitin–containing autophagosome maturation, leading to the accumulation of autophagosomes and contributing to *VCP*–pathogenesis [23], increase of FYCO1, p62 and CD63 as well as VCP supports the concept of impaired (autophagy–related) protein clearance in our in vitro system. This assumption is further supported by the fact that in *VCP*–related myopathy, VCP forms sarcoplasmic and myonuclear inclusions in IBMPFD patient tissue and the observation of cytoplasmic VCP increase in mutant fibroblasts as identified by our immunofluorescence studies. 

Further confirmational studies on muscle biopsy specimens derived from nine different *VCP*–patients revealed FYCO1 aggregation (frequently at the sub–sarcolemmal region) with partial overlap to aggregated p62, thus showing the data obtained in fibroblasts also is relevant for a tissue, skeletal muscle, which is affected by the disease. 

Given that FYCO1 increase has been linked to an acquired form of inclusion body myopathy (sIBM) [15] and that arimoclomol has been shown to have a beneficial effect on disease manifestation and progression in a mouse model of *VCP*–related inclusion body myopathy [18], we aimed to investigate the effect of arimoclomol–treatment on the fitness of a human in vitro model of the disease. Treatment of p.R155H–*VCP* mutant fibroblasts with arimoclomol indeed resulted in a profound increase of viability (mirrored by decreased cytotoxicity) in patient derived cells compared to controls as demonstrated by the results of our MTT–assay. To unravel biochemical processes which might lead to increased proliferation and reduced cytotoxicity of arimoclomol–treated *VCP*–mutant fibroblasts, we performed comparative proteomic profiling on patient derived cells with and without exposure to arimoclomol. In the treated fibroblasts, we identified increased expression of a subset of pro–survival proteins, which might accord with the increased proliferation after treatment. One of these proteins is tenascin which has already been identified to be down–regulated in *VCP*–mutant fibroblasts without arimoclomol treatment. Autophagy deficiency has been linked to blocking of tenascin degradation in turn leading to cellular cytotoxicity [24] and tenascin increase has been linked to impaired autophagic flux in *WASHC4* mutant muscle [25]. However, tenascin also promotes neurite outgrowth from cortical neurons and is implicated in guidance of migrating neurons as well as axons during development and is thus important for synaptic plasticity as well as for neuronal regeneration (https://www.uniprot.org/uniprotkb/P24821/entry (accessed on 1 February 2022)). Hence, the combined findings of increased tenascin with increased proliferation and reduced cytotoxicity in arimoclomol–treated *VCP*–mutant fibroblasts supports a beneficial role of treatment operating through a pro–survival mechanism. Along this line, it is important to note that tenascin was down–regulated in *VCP*–patient derived fibroblasts under basal conditions. Thus, arimoclomol–based increase of this protein is most likely a drug–induced compensatory mechanism toward cell survival. This assumption is supported by the concomitant increase of further pro–survival proteins (of neurological relevance) such as nestin which is required for survival, renewal and mitogen–stimulated proliferation of neural progenitor cells (https://www.uniprot.org/uniprotkb/P48681/entry (accessed on 1 February 2022)). However, further confirmational studies are needed to prove this assumption. Moreover, proteomic profiling on arimoclomol–treated cells derived from *VCP*–patients identified the increase of immune–relevant proteins such as interferon–related factors directly linked to the modulation of proteolysis, either by interaction with p62 (e.g., GBP1) or activation/modulation of E3–ubiquitin–protein ligases (e.g., DDX58) which were also up–regulated upon arimoclomol exposure in diseased fibroblasts. The increase of these proteins might reflect an influence of the drug on protein clearance which is notoriously impaired in *VCP*–etiopathology as outlined above. Our immunofluorescence–based studies toward the validation of proteomic findings obtained in arimoclomol–treated fibroblasts revealed that drug–exposure results in cytoplasmic increase of p62 in patient–derived cells. This finding might hint toward a role of p62 in protein clearance—mediated by the increase of GBP1—rather than correlating to the elevated build–up of protein aggregates upon arimoclomol treatment. This assumption is also supported by the decrease of A2MG, which is known to be able to inhibit all four classes of proteinases by a unique ‘trapping’ mechanism as well as by the results of our fitness test showing reduced cytotoxicity upon arimoclomol exposure. Focussing on the effect of arimoclomol treatment on FYCO1, our immunofluorescence studies revealed a FYCO1 increase in control fibroblasts, but not in patient fibroblasts. This finding might be related to the pathophysiological interaction of VCP with FYCO1, in turn resulting in a decrease of FYCO1 operating in autophagic flux. However, further functional studies are needed to unravel the exact biology of the pathophysiological VCP/FYCO1–interaction and its impact on efficacy of autophagic protein clearance.

## 5. Conclusions

Taken together, our combined data show that:

- FYCO1–increase also plays a role in the pathophysiology of *VCP*–related proteinopathy.
- *VCP*–patient derived fibroblasts are a suitable in vitro model to study the underlying pathophysiology.
- Arimoclomol–treatment enhances cell proliferation and viability and changes the proteomic signature of *VCP*–mutant fibroblasts toward the expression of:
  ○ Pro–survival proteins such as tenascin and nestin
  ○ Immune–relevant proteins (including interferon–modulated factors) such as GBP1, DDX58 and E3–ubiquitin–protein ligases with direct impact on proteolysis.

## Figures and Tables

**Table 1:** Patient characteristics, muscle biopsy findings and pathogenic variants of patients for which muscle biopsies were included in the study. Pat. No.: patient number; UL: upper limbs, LL: lower limbs.

Pat. No.	Sex	Age of Onset/at Biopsy	Neurological Findings at Time of Biopsy	Creatin–Kinase (U/L)	Muscle Biopsy Findings	Mono–Allelic Pathogenic Variants	Further Remarks
1	m	44/46	Scapula alata, atrophy of proximal UL, proximal paresis of LL	200–300	Muscle fiber variability, fiber splitting, focal lipofibromatosis, vacuoles, cell necrosis	c.464G>A, p.[Arg155His]	
2	m	30/35	Cramps and myalgia at muscle exercise, slight proximal paresis of LL	700–1000		c.464G>A, p.[Arg155His]	
3	m	50/58	Cramps and myalgia Proximal and distal UL weakness Distal LL weakness	432–483	Rare small angular fibers; rimmed vacuoles; rare cytoplasmic bodies, fibrosis, necrosis (rare) Type 1 fiber predominance	c.648A>G p.[Ile216Met]	No family history
4	f	43/45	Scapula alata; distal UL and LL weakness	Normal	Fiber size variability; small atrophic fibers; some internalized nuclei; rimmed vacuoles; ragged red fibers (rare); Type 1 fiber predominance	c.474G>A p.[Met158Ile]	Scoliosis from childhood
5	m	60/63	Scapula alata; Proximal UL weakness; Distal UL and LL weakness (drop feet)	317	Slight fiber size variability; some internalized nuclei; scattered small atrophic fibers; focal increase of connective tissue	c.785C>G p.[Thr262Ser]	
6	m	50/53	Scapula alata Proximal UL, distal LL weakness	557	Moderate fiber size variability; some internalized nuclei; scattered small atrophic fibers; focal increase of connective tissue, Rimmed vacuoles	c.464G>A, p.[Arg155His]	Scoliosis, Paget’s bone disease
7	M	69/71	Scapula alata, proximal UL and LL, distal LL weakness	230	Slight fiber size variability; some internalized nuclei; scattered small atrophic fibers; focal increase of connective tissue	c.C277C>T, p.[Arg93Cys]	Scoliosis, axonal neuropathy of the legs

**Table 2:** List of up– and downregulated proteins in *VCP*–mutant fibroblasts treated with arimoclomol. The term entry reflects UniProt identification number.

Accession	Protein Name	Fold Change	*p*–Value	Function
Upregulated upon Arimoclomol–Treatment				
O95786	Probable ATP–dependent RNA helicase DDX58	8.10	0.039	activates expression of IFN–alpha and IFN–beta interferons and recruits E3 ubiquitin–protein ligases
O14879	Interferon–induced protein with tetratricopeptide repeats 3	7.40	0.020	can negatively regulate the apoptotic effects
P32455	Guanylate–binding protein 1	5.55	0.008	interaction with ubiquitin–binding protein SQSTM1, which delivers monoubiquitylated proteins to autolysosomes
Q15043	Zinc transporter ZIP14	4.47	0.001	regulates insulin receptor signalling, glucose uptake, glycogen synthesis and gluconeogenesis
Q03519	Antigen peptide transporter 2	3.97	0.006	typically transports intracellular peptide antigens of 8 to 13 amino acids that arise from cytosolic proteolysis via IFNG–induced immunoproteasome
P61601	Neurocalcin–delta	3.81	0.041	calcium–dependent regulation of rhodopsin phosphorylation
P31350	Ribonucleoside–diphosphate reductase subunit M2	3.74	0.001	catalyzes the biosynthesis of deoxyribonucleotides from the corresponding ribonucleotides and inhibits Wnt signaling
Q460N5	Poly [ADP–ribose] polymerase 14	3.72	0.010	regulates pro–inflammatory cytokine production in macrophages in response to IFNG stimulation
P02794	Ferritin heavy chain	3.32	0.000	stores iron in a soluble, non–toxic, readily available form
O95379	Tumor necrosis factor alpha–induced protein 8	3.23	0.004	suppresses the TNF–mediated apoptosis by inhibiting caspase–8 activity
Q8IYM9	E3 ubiquitin–protein ligase TRIM22	2.97	0.003	interferon–induced antiviral protein with E3 ubiquitin–protein ligase activity
P24821	Tenascin	2.85	0.001	implicated in guidance of migrating neurons as well as axons during development, synaptic plasticity as well as neuronal regeneration
Q9Y508	E3 ubiquitin–protein ligase RNF114	2.81	0.000	E3 ubiquitin–protein ligase that promotes the ubiquitination of various substrates
P48681	Nestin	2.56	0.045	required for survival, renewal and mitogen–stimulated proliferation of neural progenitor cells
P28838	Cytosol aminopeptidase	2.47	0.002	involved in the metabolism of glutathione and in the degradation of glutathione S–conjugates,
Q8TCS8	Polyribonucleotide nucleotidyltransferase 1, mitochondrial	2.45	0.003	plays a role in mitochondrial morphogenesis and respiration; regulates the expression of the electron transport chain (ETC) components at the mRNA and protein levels
Q9NR77	Peroxisomal membrane protein 2	2.42	0.008	contributes to the unspecific permeability of the peroxisomal membrane
Q06330	Recombining binding protein suppressor of hairless	2.34	0.041	transcriptional regulator that plays a central role in Notch signaling
Q9BQE5	Apolipoprotein L2	2.33	0.000	modulates movement of lipids in the cytoplasm or allows the binding of lipids to organelles
P01116	GTPase KRas	2.25	0.005	plays an important role in the regulation of cell proliferation
P41226	Ubiquitin–like modifier–activating enzyme 7	2.21	0.001	activates ubiquitin by first adenylating with ATP its C–terminal glycine residue
Q9Y547	Intraflagellar transport protein 25 homolog	2.18	0.011	component of the IFT complex B required for sonic hedgehog/SHH signaling
Q99836	Myeloid differentiation primary response protein MyD88	2.16	0.006	adapter protein involved in the Toll–like receptor and IL–1 receptor signaling pathway in the innate immune response
Q5D862	Filaggrin–2	2.15	0.007	essential for normal cell–cell adhesion in the cornified cell layers
Q9P258	Protein RCC2	2.15	0.001	multifunctional protein that may affect its functions by regulating the activity of small GTPases, such as RAC1 and RALA
O94808	Glutamine––fructose–6–phosphate aminotransferase [isomerizing] 2	2.14	0.015	controls the flux of glucose into the hexosamine pathway
Q96T88	E3 ubiquitin–protein ligase UHRF1	2.14	0.025	E3 ubiquitin–protein ligase activity by mediating the ubiquitination of target proteins
P19474	E3 ubiquitin–protein ligase TRIM21	2.09	0.000	E3 ubiquitin–protein ligase whose activity is dependent on E2 enzymes
P33908	Mannosyl–oligosaccharide 1,2–alpha–mannosidase IA	2.07	0.016	involved in the maturation of Asn–linked oligosaccharides
Q08AF3	Schlafen family member 5	2.07	0.002	plays a role in hematopoietic cell differentiation
P52434	DNA–directed RNA polymerases I, II, and III subunit RPABC3	2.03	0.003	DNA–dependent RNA polymerase catalyzes the transcription of DNA into RNA
P54105	Methylosome subunit pICln	2.02	0.021	chaperone that regulates the assembly of spliceosomal U1, U2, U4 and U5 small nuclear ribonucleoproteins
O43598	2’–deoxynucleoside 5’–phosphate N–hydrolase 1	2.02	0.001	catalyzes the cleavage of the N–glycosidic bond of deoxyribonucleoside 5’–monophosphates to yield deoxyribose 5–phosphate
O60879	Protein diaphanous homolog 2	2.02	0.006	involved in the regulation of endosome dynamics
Q8NFH5	Nucleoporin NUP35	2.01	0.000	functions as a component of the nuclear pore complex
**Downregulated upon arimoclomol–treatment**				
Q7Z7M9	Polypeptide N–acetylgalactosaminyltransferase 5	0.50	0.001	catalyzes the initial reaction in O–linked oligosaccharide biosynthesis
Q16706	Alpha–mannosidase 2	0.49	0.000	catalyzes the first committed step in the biosynthesis of complex N–glycans
P02511	Alpha–crystallin B chain	0.48	0.036	has chaperone–like activity, preventing aggregation of various proteins under a wide range of stress conditions
Q9Y2G5	GDP–fucose protein O–fucosyltransferase 2	0.47	0.000	catalyzes the reaction that attaches fucose through an O–glycosidic linkage to a conserved serine or threonine residue
O14494	Phospholipid phosphatase 1	0.44	0.001	regulates phospholipid–mediated signaling pathways
O60637	Tetraspanin–3	0.32	0.002	regulates the proliferation and migration of oligodendrocytes
P01023	Alpha–2–macroglobulin	0.19	0.045	inhibits all four classes of proteinases by a unique ‘trapping’ mechanism

## Data Availability

The proteomic profiling data have been deposited in the ProteomeXchange Consortium via the PRIDE partner repository with the dataset identifier PXD.

## Supplementary Materials

The following supporting information can be downloaded at: https://www.mdpi.com/article/10.3390/biomedicines10102443/s1, A list of all proteins dysregulated in *VCP*–patient derived fibroblasts under basal conditions can be found in the supplement (Supplementary Table S1). Results of the MRI studies are presented in the Supplementary Figure S1 whereas Supplementary Figure S2 shows the Volcano–plot referring to the proteomic profile obtained on *VCP*–mutant fibroblasts in comparison to controls.
