# Supplementary material for: FYCO1 Increase and Effect of Arimoclomol–Treatment in Human VCP–Pathology
Source: Biomedicines. 2022 Sep 30;10(10):2443. doi: 10.3390/biomedicines10102443 (PMC9598455; doi:10.3390/biomedicines10102443)
Supplement: Supplementary file 1 [file biomedicines-10-02443-s001.zip › legends_to_supplementary figures.pdf]

Figure legends to supplementary figures

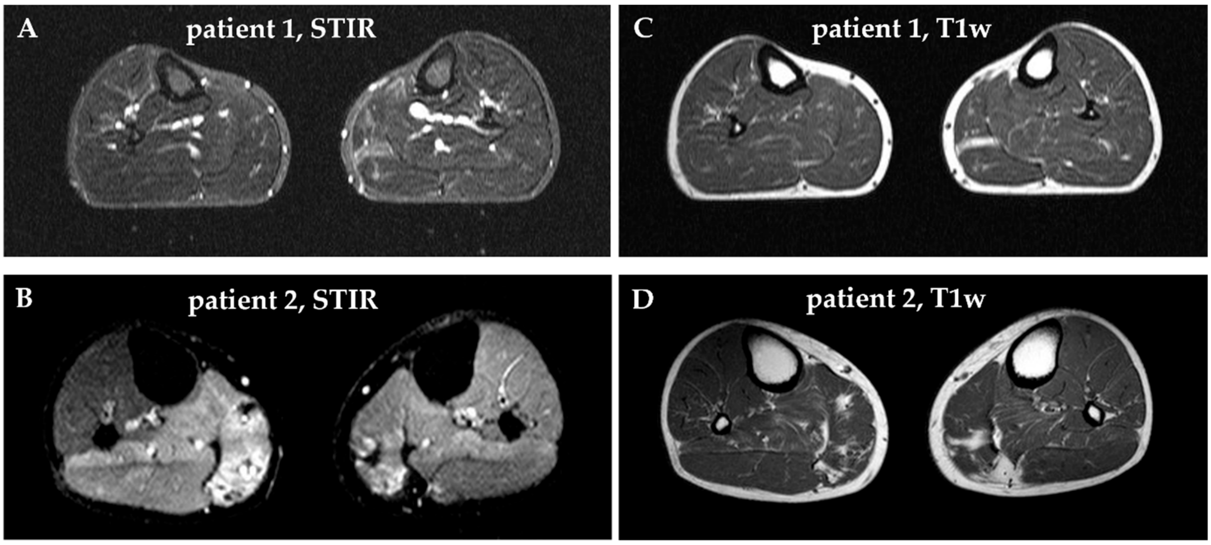

Supplementary Figure S1. Skeletal muscle MRI of the calfs of 2 *VCP*-patients. (**A**) and (**B**) show edema in the STIR-sequences, (**C**) and (**D**) display fatty degeneration in T1w-sequences. STIR: short-tau-inverted-recovery-sequence.

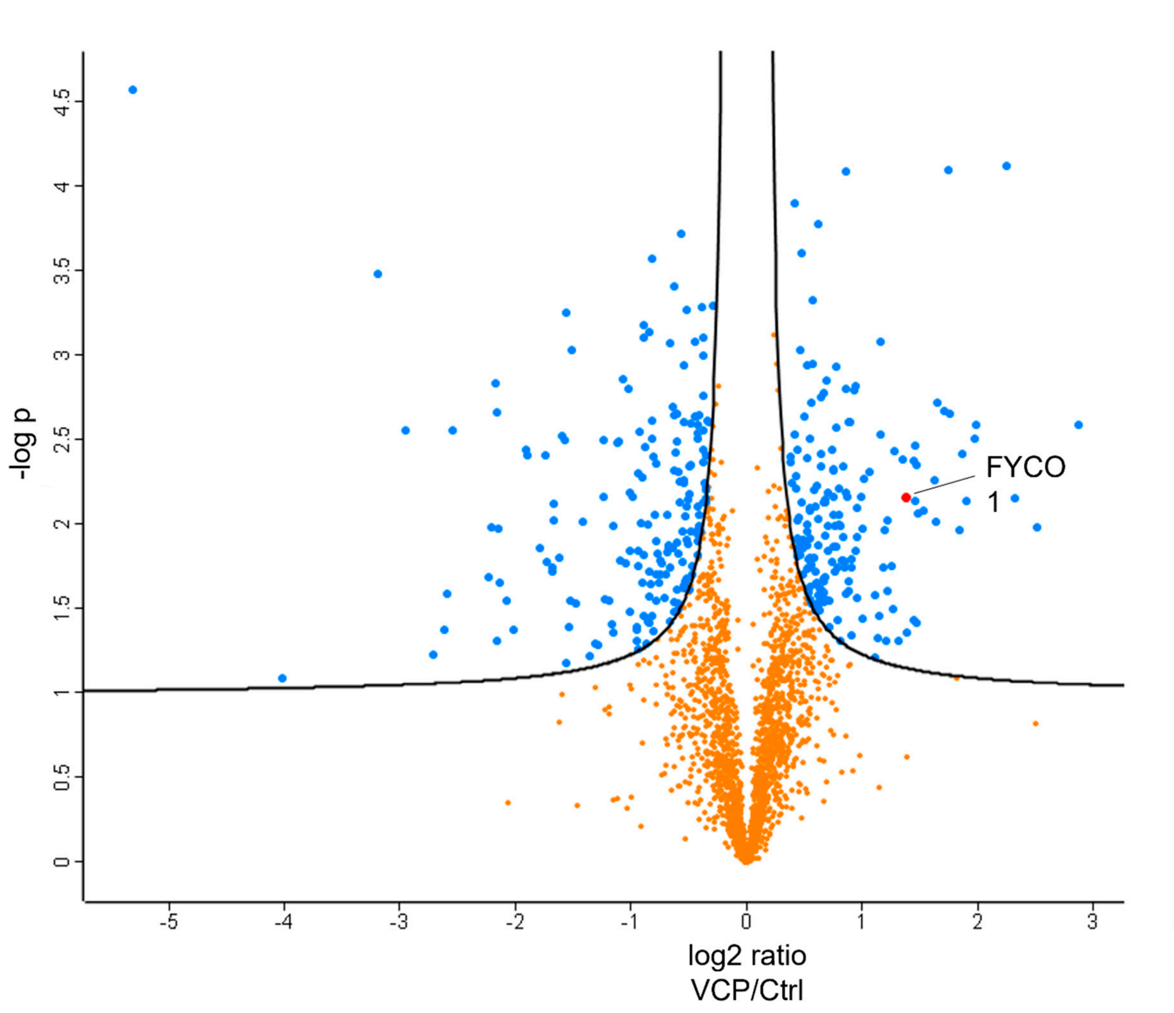

Supplementary Figure S2: Volcano plot of proteomic data obtained on VCP-patient derived fibroblasts; blue dots representing significantly regulated proteins with an adjusted p-value <0.05. (done with Perseus). Red dot highlights the statistically significant increase of FYCO1 as well as the statistically significant decrease of tenascin.
