# Supplementary figures and images for: FYCO1 Increase and Effect of Arimoclomol–Treatment in Human VCP–Pathology
Source: Biomedicines. 2022 Sep 30;10(10):2443. doi: 10.3390/biomedicines10102443 (PMC9598455; doi:10.3390/biomedicines10102443)

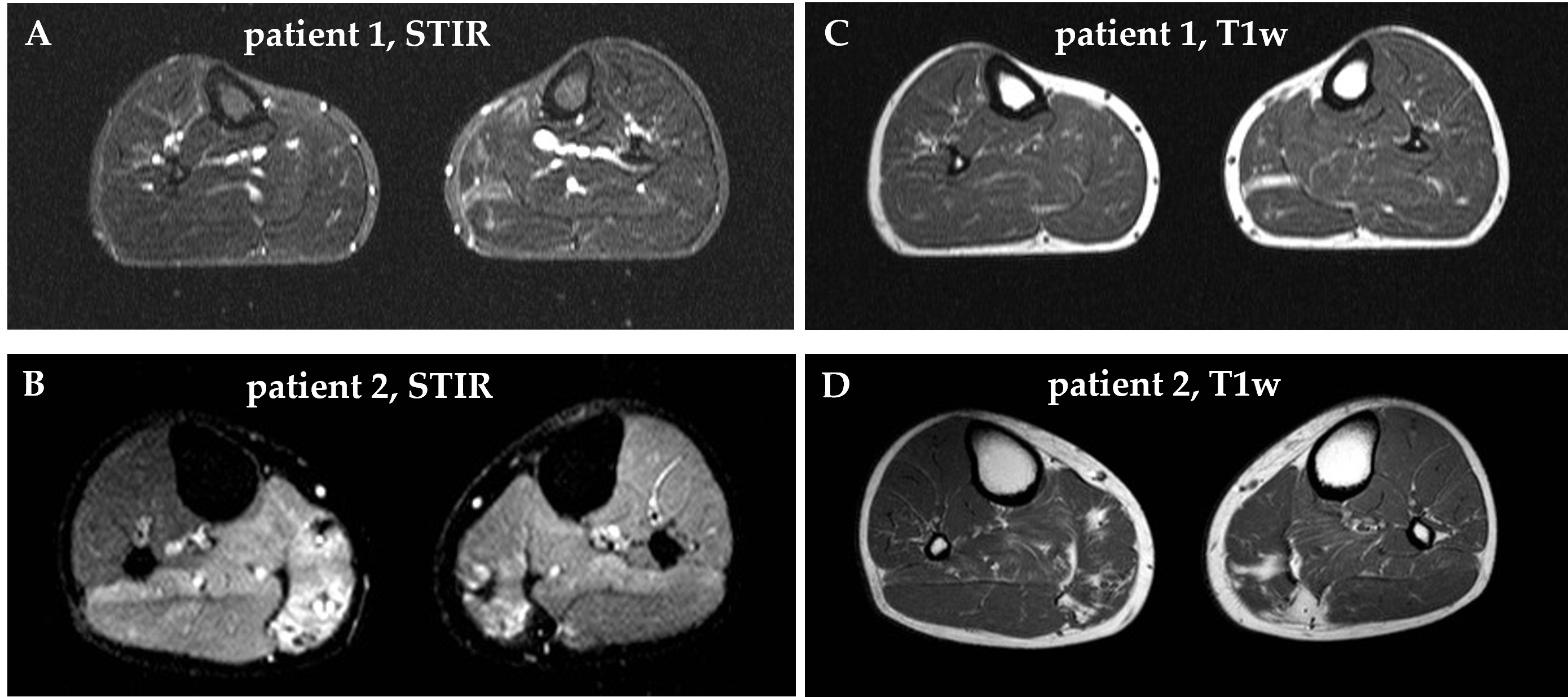

Supplement: Supplementary file 1 [file biomedicines-10-02443-s001.zip › supplementary figure 1.tif]

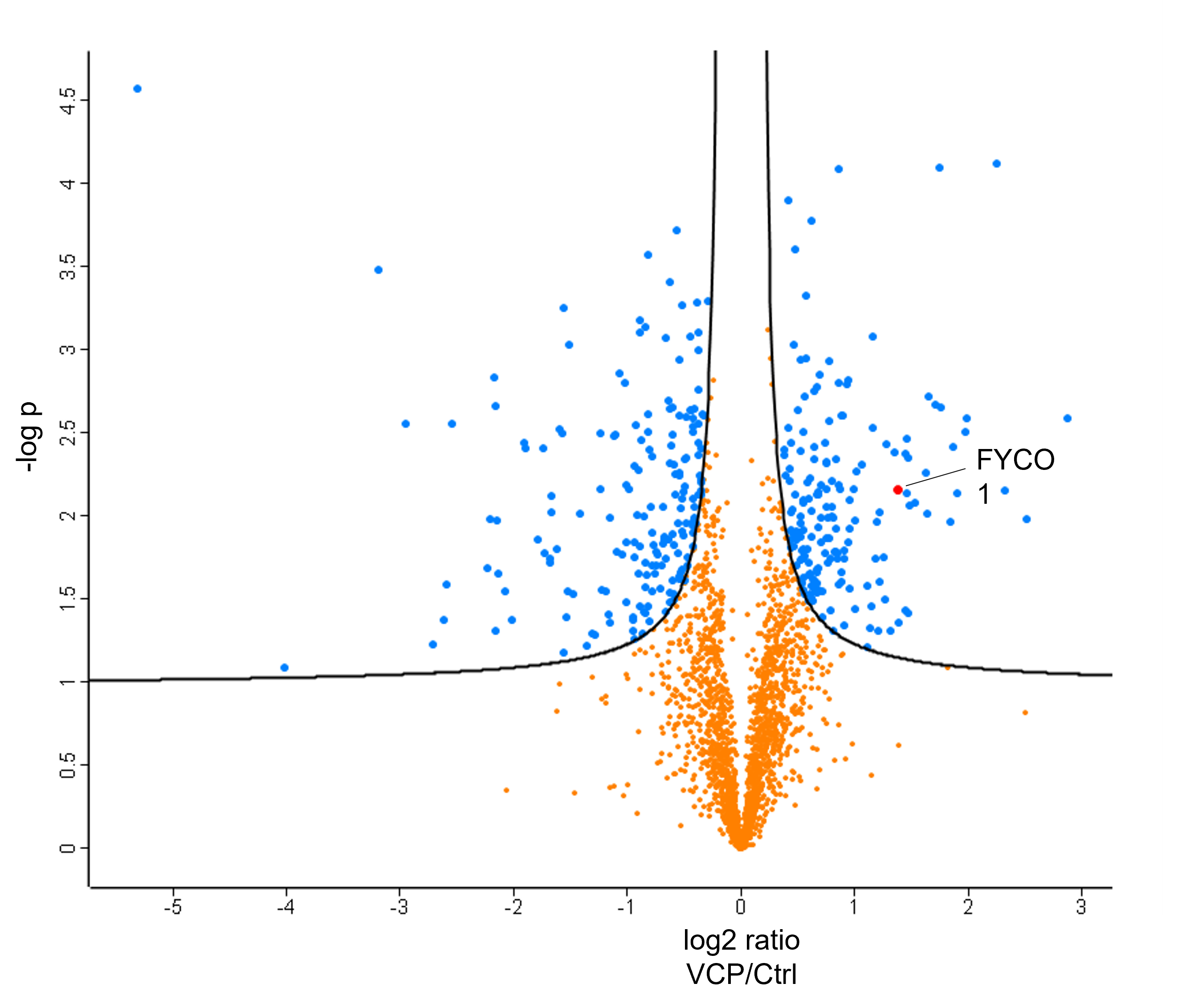

Supplement: Supplementary file 1 [file biomedicines-10-02443-s001.zip › supplementary figure 2.tif]
